# New species of *Caromiobenella* Jeon, Lee & Soh, 2018 (Crustacea, Copepoda, Monstrilloida) from Chuja Island, Korea

**DOI:** 10.3897/zookeys.814.29126

**Published:** 2019-01-08

**Authors:** Donggu Jeon, Wonchoel Lee, Ho Young Soh

**Affiliations:** 1 Department of Life Science, College of Natural Sciences, Hanyang University, Seoul, South Korea Hanyang University Seoul Korea, South; 2 Faculty of Marine Technology, College of Fisheries and Ocean Science, Chonnam National University, Yeosu, Jeollanam-do, South Korea Chonnam National University Yeosu Korea, South

**Keywords:** *Caromiobenellaohtsukai* sp. n., molecular phylogeny, planktonic copepod, taxonomy

## Abstract

Male monstrilloid copepods belonging to the genus *Caromiobenella* Jeon, Lee & Soh, 2018 were collected from Chuja Island, Jeju, Korea, using a light trap. This paper describes a new species, *Caromiobenellaohtsukai***sp. n.**, based on the display of reduced, knob-like fifth legs on the ventral side of the first urosomal somite. A unique combination of male genitalia features and number of caudal setae further confirms its specificity. Molecular analysis based on two partial gene sequences of mitochondrial cytochrome *c* oxidase subunit I (mtCOI) and 28S ribosomal RNA (28S rRNA) also supports the designation of this species by showing a relevant divergence from known congeners. *Caromiobenellaohtsukai***sp. n.** is the ninth member of this genus and also the ninth monstrilloid reported from Korea.

## Introduction

Monstrilloids are semi-parasitic copepods with a complex life cycle that includes an endoparasitic juvenile phase and a free-living planktonic adult phase. The early infective nauplii are also planktonic, but soon infect hosts. Juveniles are known to infect various marine invertebrates such as polychaetes, mollusks, and sponges (Boxshall and Halsey 2004; Huys et al. 2007; Suárez-Morales et al. 2014). Adults are generally scarce in open marine water, but are known to occur frequently in reef environments, especially at nighttime (Sale et al. 1976; Sekiguchi 1982; Suárez-Morales 2001; Grygier and Ohtsuka 2008). The order Monstrilloida Sars, 1901 currently contains a single family, Monstrillidae Dana, 1849, consisting of about 160 nominal species in six valid genera: *Monstrilla* Dana, 1849; *Cymbasoma* Thompson, 1888; *Monstrillopsis* Sars, 1921; *Maemonstrilla* Grygier & Ohtsuka, 2008; *Australomonstrillopsis* Suárez-Morales & McKinnon, 2016; and *Caromiobenella* Jeon, Lee & Soh, 2018.

Sars (1921) described *Monstrillaserricornis* based on two Norwegian male specimens collected from Bukken (outside the Stavanger Fjord) and Kvalø (the Nordland coast). The males were, at that time, characterized by the display of a modified last antennular segment reportedly armed with “five small recurved denticles” distomedially. In addition to this unusual modification, other ambiguous features such as a relatively short cephalothorax and a poorly developed oral papilla led Sars (1921) to provisionally assign this species to *Monstrilla*. McAlice (1985) reexamined Sars’ Bukken specimen and confirmed that the “denticles” on the last antennular segment were actually transverse rows, each consisting of numerous fine spines or setules. Later, Huys and Boxshall (1991) defined four types of male antennule in the order Monstrilloida: type 1 lacks prominent modifications on the distal segment and is found in most *Monstrilla* and *Cymbasoma* species; type 2 has the distal segment with an inner proximal hyaline bump and a distal part gradually tapering and slightly curved inwards and is found in *Monstrillopsis* species; type 3 is characterized by the presence of five transverse rows on the distal segment, as explained above; type 4 is similar to type 3 but the marginal rows are much reduced and is only observed in *Cymbasomalongispinosum* (Bourne, 1890). Previous authors also recognized that the genus *Monstrilla* included several species in which males presented type 3 antennules, and that those species form a small group within the genus. The type 3 antennular modification, however, had not been considered to be a genus-distinguishing feature, whereas type 2 morphology is exclusive of male *Monstrillopsis*. Suárez-Morales et al. (2008) also confirmed that type 3 antennules are shared several species including *M.helgolandica*, *M.serricornis*, *M.pygmaea*, and *M.patagonica*. Jeon et al. (2018a) found that species with type 3 antennules are different morphologically and genetically from the majority of *Monstrilla* species with type 1 antennules, and consequently established the genus *Caromiobenella* for eight species with type 3 antennules: *C.helgolandica* (Claus, 1863); *C.serricornis* (Sars, 1921); *C.arctica* (Davis & Green, 1974); *C.hamatapex* (Grygier & Ohtsuka, 1995); *C.pygmaea* (Suárez-Morales, 2000); *C.patagonica* (Suárez-Morales, Ramírez & Derisio, 2008); *C.castorea* Jeon, Lee & Soh, 2018 (type species), and *C.polluxea* Jeon, Lee & Soh, 2018. This study provides a taxonomic account of a new species of *Caromiobenella*. Results presented herein support the validity and supplement the initial description of the genus *Caromiobenella* and its species diversity.

## Materials and methods

### Sample collection and preparation for morphological analysis

Samples were collected with a hand-made light trap (a PVC pipe, 400 mm long and 100 mm in diameter) containing a LED flash light. Contents attracted to the trap was filtered through a 63 μm mesh test sieve, and the retained material was immediately washed several times with 99.5% ethanol. Samples were initially fixed with 99.5% ethanol at the collection site and transferred to fresh 99.5% ethanol in the laboratory. Monstrilloids were isolated from the bulk collection of specimens with aid of a SMZ645 stereomicroscope (Nikon, Tokyo, Japan) and kept refrigerated at 4 °C.

Monstrilloid copepods used for morphological descriptions were treated with 0.5% sodium phosphate tribasic dodecahydrate solution (Na_3_PO_4_∙12H_2_O; Daejung Chemicals & Metals, Siheung, Korea) to restore their original shape (Van Cleave and Ross 1947; Huys and Boxshall 1991; Jeon et al. 2018a). Drawings were prepared using an Eclipse 80i compound microscope (Nikon) equipped with differential interference contrast optics and a drawing tube. The specimens were dissected to small parts after the habitus observation, and each part was mounted on a glass slide with lactophenol for detailed microscopic examination. Body measurements were acquired using AxioVision LE64 software (AxioVs40x64v 4.9.1.0; Carl Zeiss, Oberkochen, Germany). Length of each body somite was measured along dorsal medial line from the anterior end to the posterior end, and total body length was represented by a sum of those measurements. Terminology from Grygier and Ohtsuka (2008) and Jeon et al. (2018b) was used to describe body segmentation and antennular setation patterns, respectively. For caudal setae numbering, definitions from Huys and Boxshall (1991) were adopted over those of Huys et al. (2007) in consideration of the seemingly dorsal position of the VII seta.

### Molecular analysis

Genomic DNA (gDNA) extraction using Chelex 100 chelating resin (molecular biology grade, 200–400 mesh, sodium form; Bio-Rad, Hercules, CA, USA) was carried out according to methods outlined in previous studies (Estoup et al. 1996; Casquet et al. 2012). Portions of mtCOI and 28S rRNA genes were amplified using an AccuPower HotStart PCR PreMix kit (Bioneer, Daejeon, Korea). Each reaction tube for the amplification was prepared with 16 μl of distilled water, 2 μl of gDNA template, and 1 μl each of the forward and reverse primers. Thermal cycling was performed using Mastercycler (Eppendorf, Hamburg, Germany) or MyGenie 96 Thermal Block (Bioneer). For mtCOI gene amplification, XcoiF (5’-ATAACRCTGTAGTAACTKCTCAYGC-3’; Jeon et al. 2018b) and HCO2198 (5’-TAAACTTCAGGGTGACCAAAAAATCA-3’; Folmer et al. 1994) primers were used. The thermal cycling profile was 5 min at 94 °C for initial denaturation followed by 40 sec at 94 °C for denaturation, 45 sec at 50 °C for annealing, and 45 sec at 72 °C for elongation. This reaction profile was repeated 40 times, followed by a final 7 min elongation step at 72 °C. To amplify the 28S rRNA gene, 28S-F1a (5’-GCGGAGGAAAAGAAACTAAC-3’) and 28S-R1a (5’-GCATAGTTTCACCATCTTTCGGG-3’) primers were used (Ortman 2008). The thermal cycling profile was 5 min at 94 °C for initial denaturation followed by 1 min at 94 °C for denaturation, 1 min at 50 °C for annealing, and 1 min at 72 °C for elongation. This reaction profile was repeated 35 times, followed by a final 7 min elongation step at 72 °C. PCR products were run on a 1% Tris acetate-EDTA agarose gel for 20 min at a voltage of 100 V with a 100 bp DNA ladder (Bioneer). PCR products with positive results were sent to Macrogen (Seoul, Korea) for purification and DNA sequencing.

Sequencing chromatograms were read using FinchTV software (ver. 1.4.0). Sequences were further edited using MEGA7 software (ver. 7.0.21; Kumar et al. 2016) to exclude both forward and reverse primer sequences. Sequences were aligned using ClustalW implemented in MEGA7. Genetic divergences among and within species were calculated under the Kimura 2-parameter model (K2P) with 3,000 bootstrapping replicates. Substitution saturation was tested for both sets of mtCOI and 28S rRNA sequences using DAMBE software (ver. 7.0.28; Xia et al. 2003; Xia and Lemey 2009). Maximum Likelihood (ML) phylogenetic trees were generated with MEGA7, and Bayesian Inference (BI) phylogenetic trees were generated with MrBayes (ver. 3.2.6; Ronquist et al. 2012). The best-fit models for ML analysis were searched based on the corrected Akaike Information Criteria (AICc) using jModelTest (ver. 2.1.10; Darriba et al. 2012). For mtCOI, TIM1+I+G (1st), TIM1+G (2nd), and GTR+I+G (3rd) were selected; and for 28S rRNA, TIM3+G (1st), TIM3+I+G, and GTR+G (3rd) were selected. For ML and BI tree reconstructions, we used the third best-fit substitution model for each analysis (i.e., GTR+I+G for mtCOI and GTR+G for 28S rRNA) because other than those two models were not compatible with MEGA7 and MrBayes. The ML analysis was carried out with 3,000 bootstrapping replicates. The BI analysis was run for 5,000,000 generations sampling every 100 generations, and the first 25% of the generations were discarded before the final tree reconstruction. Partitioned Bayesian analysis for combined mtCOI and 28S rRNA sequences was conducted by applying the best-fit model (described above) for each gene region.

## Results

### Systematics

#### Order Monstrilloida Sars, 1901

##### Family Monstrillidae Dana, 1849

###### Genus *Caromiobenella* Jeon, Lee & Soh, 2018

####### Caromiobenella ohtsukai sp. n.

Taxon classificationAnimaliaMonstrilloidaMonstrillidae

http://zoobank.org/3815AE9D-184C-49C5-BC2D-90CF969D526C

Figs 1 , 2 , 3 , 4

######## Type locality.

Yeongheung-ri (33°57.59”N; 126°17.82”E), Chuja-myeon, Jeju-si, Jeju-do, Republic of Korea. English equivalents of political divisions in Korea: ri = village; myeon = township; si = city; do = province.

######## Type material examined.

Specimens were collected by Dr Min Ho Seo (Marine Ecology Research Center, Korea) using a light trap on 11 September 2017 alongside a small harbor at the type locality. The depth at the sampling site was about 3 m. The type specimens are deposited in the National Marine Biodiversity Institute of Korea (MABIK), Seocheon, Korea, with the following accession numbers: male holotype (MABIK CR00244260) dissected and mounted on five slides in lactophenol; six intact male paratypes (MABIK CR00244261) in 99.5% ethanol vial. Five additional specimens were used for molecular analysis.

######## Species diagnosis

**(male).** Total body length 1.12–1.30 mm (mean 1.21; *N* = 7). Length ratio (lateral view) of cephalothorax: metasome: urosome as 33.1 (32.1–35.7): 40.9 (39.3–42.0): 26.0 (25.0–27.6). Oral papilla inconspicuous, rather retracted inwards, located ventrally within 27.3% (25.1–31.6) of distance from anterior margin of cephalothorax. Length of antennules in relation to total body length 30.2% (27.7–33.8), to cephalothorax length 90.4% (82.8–95.2). Antennular segments relative length (as % of antennule total length) from proximal to distal as 15.7 (14.8–17.4): 19.8 (18.7–20.8): 18.2 (16.6–19.3): 23.0 (21.5–24.9): 23.4 (21.5–24.3). Distal segment lacking branched setae, with unbranched simple setae instead. Fourth segment with prominent accessory spine 4a on inner dorsodistal margin. Distal margin of intercoxal sclerite of legs 1–4 excavated. First urosomal somite with extremely reduced, knob-like fifth legs devoid of setae inserted on posteroventral margin. Genital shaft 0.07 (0.068–0.079) mm long, basal half robust, distal half gradually tapering; proximal and distal parts distinguished by anterior protrusion in lateral view; distalmost part with smooth medial protrusion; two subtriangular lappets arising from distolateral sides of shaft, span of lappets not exceeding width of succeeding postgenital somite. Genital opercular openings covered by hand-like opercular flaps placed at distal end of genital shaft. Caudal rami with 6 setae (II–VII); dorsal apical seta VII conspicuously shorter than rest.

######## Description of male holotype.

**Figure 1.**
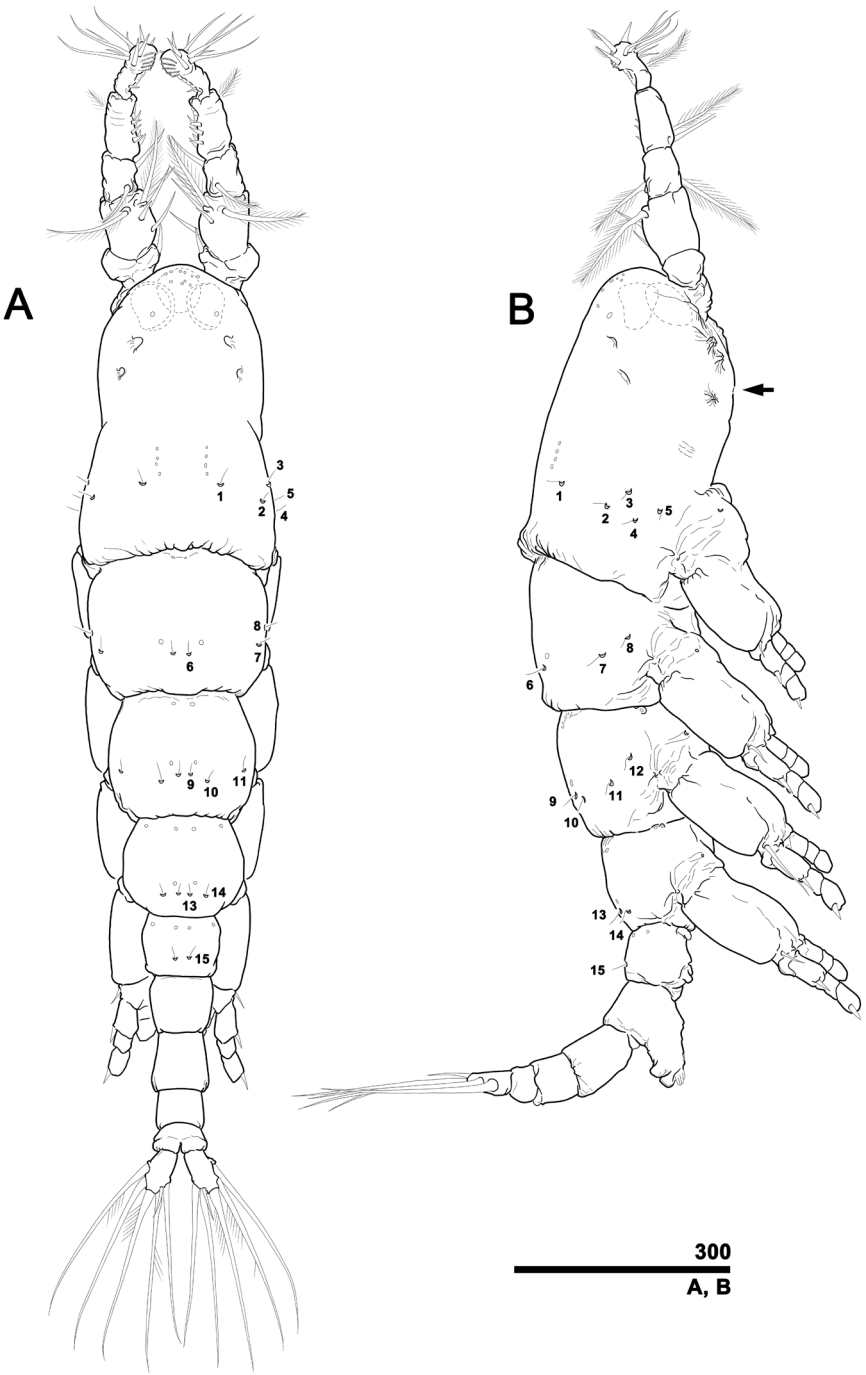
*Caromiobenellaohtsukai* sp. n., male holotype (MABIK CR00244260) **A** Habitus in dorsal view with pit-setae 1–15 of right side indicated **B** Habitus in lateral view with poorly developed oral papilla indicated (arrow). Two lateral and one ventral eyes are indicated using dotted-lines (cf. Fig. 1A, B). Scale bar in micrometers.

Total body length excluding antennules and caudal rami 1.24 mm in dorsal view, 1.25 mm in lateral view. Body consisting of nine somites: cephalothorax incorporating first pedigerous somite, free somites 1–3, first urosomal somite, genital somite, postgenital somite, penultimate somite, and anal somite (Fig. 1A, B). Length of somites as percentage of total body length: 32: 16: 14: 11: 7: 6: 6: 4: 3 in dorsal view; 35: 17: 15: 11: 6: 6: 6: 4: 2 in lateral view.

**Figure 2.**
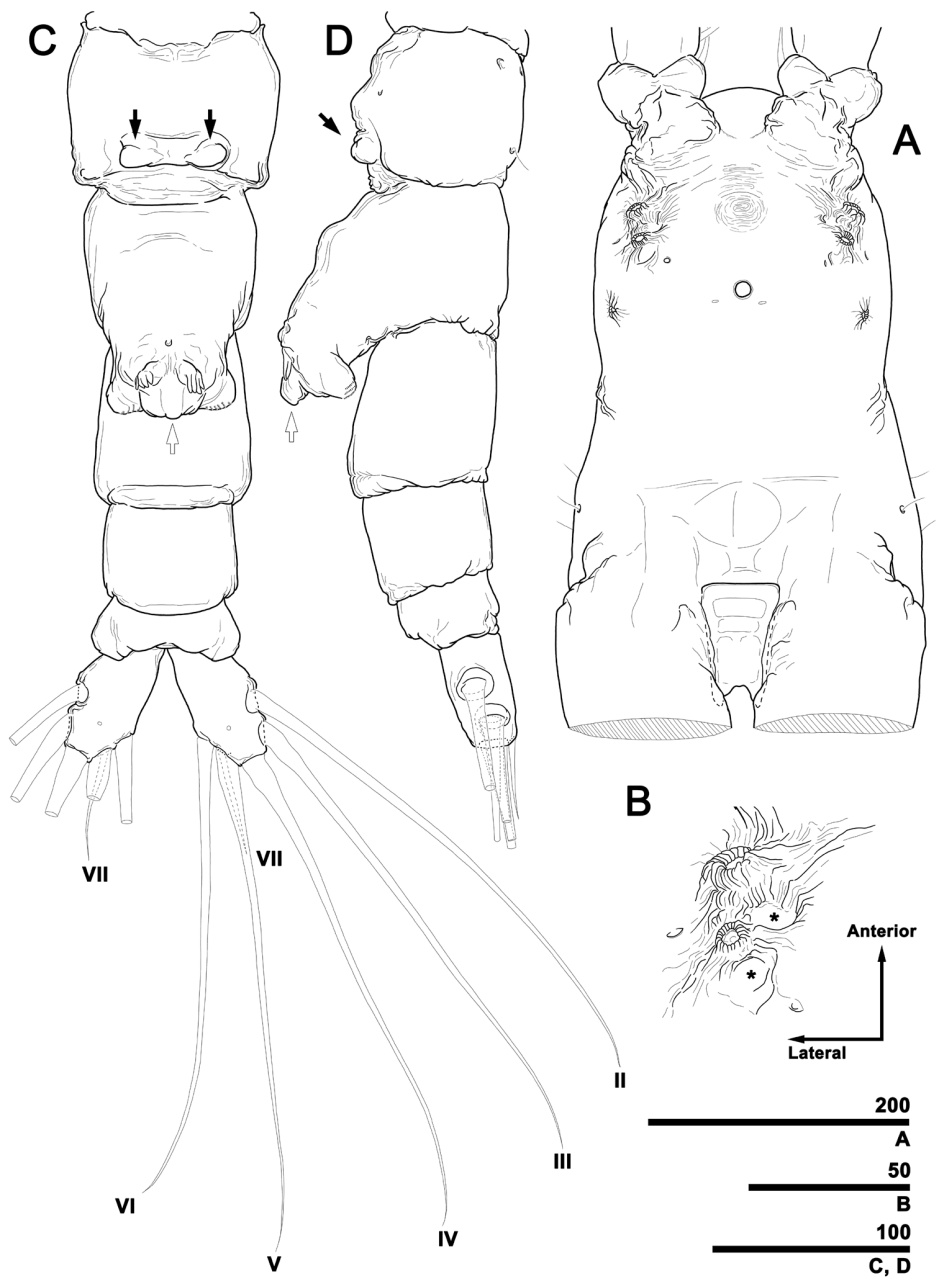
*Caromiobenellaohtsukai* sp. n., male holotype (MABIK CR00244260) **A** Cephalothorax, ventral **B** Two anterior scars on right side, each followed by rounded depression (asterisks) **C** Urosome, ventral, showing small, knob-like fifth legs (black arrows), medial protrusion on genital apparatus (white arrow), and caudal setae (II–VII) **D** Urosome, lateral, showing small knob-like fifth legs (black arrow) and medial protrusion on genital apparatus (white arrow). Scale bars in micrometers.

Cephalothorax incorporating first pediger rather short, 0.40 mm long in dorsal view, 0.43 mm in lateral view, generally bullet-shaped in dorsal view. Anterior margin convex, without typical forehead sensilla. Length 1.2 times greater than maximal width, narrowest (0.23 mm) at 58.6% of distance from anterior margin. Width of incorporated first pediger 0.37 mm near posterior margin (at 91.6% of distance from anterior margin), this being widest part of cephalothorax. Anterodorsal part of cephalothorax with several pores (Fig. 1A, B). Two pairs of concave depressions posterior to porous region, with anterior pair located closer to central body axis than posterior pair. Ventral side of cephalothorax with three pairs of scars (Fig. 2A): two prominent pairs posterior to antennular bases, and relatively inconspicuous pair placed more laterally at one-third length of cephalothorax. Anteriormost and posteriorly adjacent scars each followed by rounded depression (Fig. 2B). Oral papilla situated ventrally between posteriormost pair of scars, not protrusive at all, almost rudimentary, retracted in lateral view (Fig. 1B). Two pores located behind oral papilla. Cuticle of ventromedial region between two anterior pairs of scars with fingerprint-like whirling pattern with fine wrinkles (Fig. 2A). Tergite of incorporated first pediger with five pairs of pit-setae (Fig. 1A, B): one pair (no. 1) situated dorsally, four pairs laterally (no. 2–5). Two longitudinal rows of four faint pores each located slightly anteriorly to pit-setae 1, arranged in parallel across midline.

Two lateral and one ventral eyes (Fig. 1A, B) placed at anterior quarter of cephalothorax, all moderately developed and pigmented. Ventral eye positioned slightly anterior to lateral eyes. Lateral eyes bean-shaped in dorsal view, 64.8 μm in diameter, placed 0.02 mm apart across midline. Ventral eye round in dorsal view, but oval in lateral view. Ventral eye slightly smaller in diameter (60.1 μm) than lateral counterparts in dorsal view.

First free pedigerous somite to first urosomal somite each with several pore pairs in various regions (Fig. 1A, B). First free somite with three pairs of pit-setae posteriorly (no. 6–8: pair dorsally, two pairs laterally), plus pair of simple pores anterior to dorsal pair of pit-setae. Second free somite with four pairs of pit-setae posteriorly (no. 9–12: two pairs dorsally, other two pairs laterally), plus pair of simple pores anterior to dorsal pair of pit-seta. Third free somite with two pairs of pit-setae posteriorly (no. 13, 14), all aligned transversally across dorsum, plus pair of simple pores anterior to them. First urosomal somite with pair of pit-setae (no. 15) on posterior dorsal surface. Each free somite also with one or two pairs of pores in anterior dorsum.

**Figure 3.**
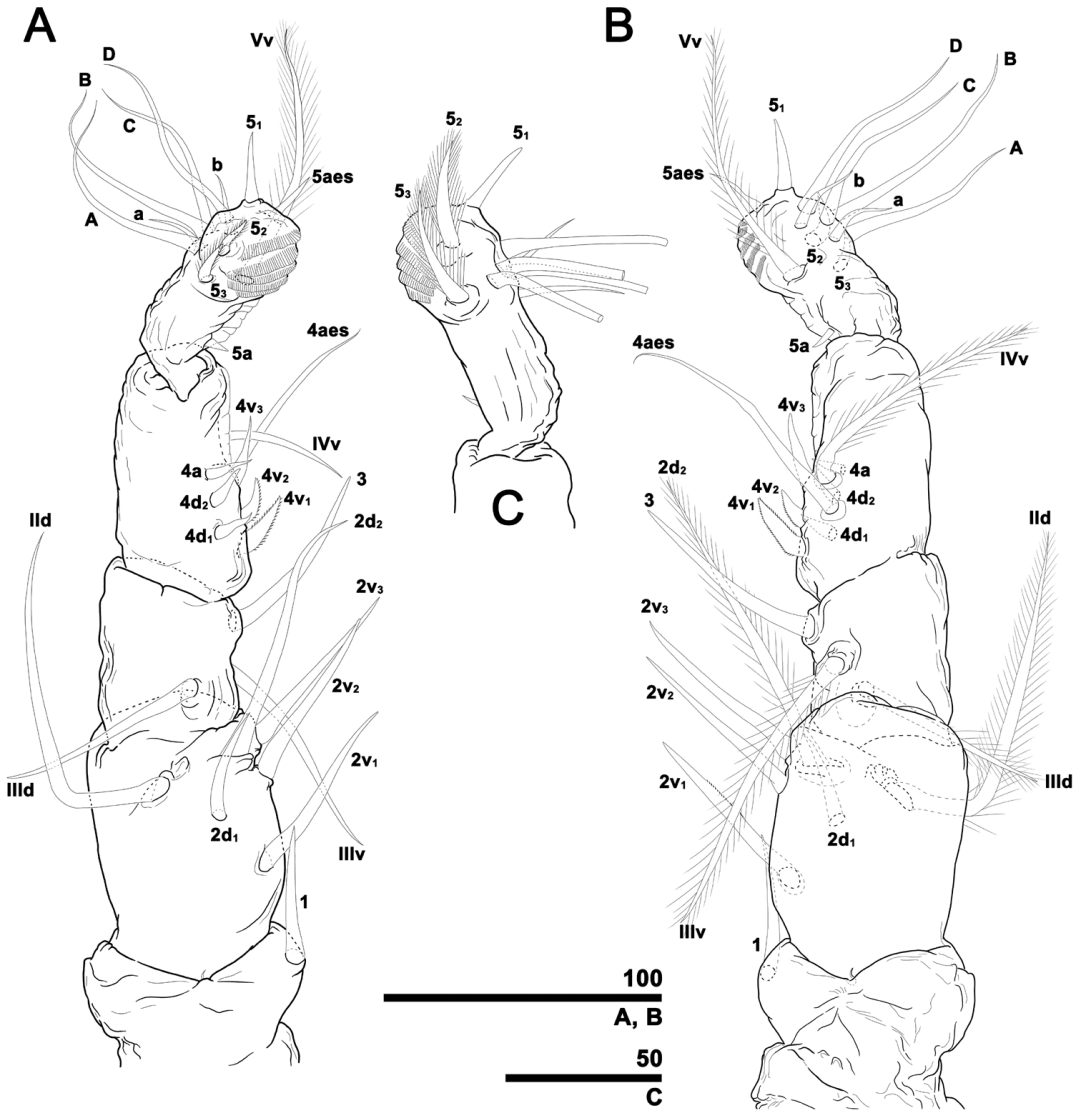
*Caromiobenellaohtsukai* sp. n., male holotype (MABIK CR00244260), antennule labelling following Jeon et al. (2018b)**A** Left antennule, dorsal, last segment slightly upward (cf. Fig. 3C) **B** Left antennule, ventral, last segment slightly downward (cf. Fig. 3C) **C** Right antennule, last segment showing actual length of spines 5_2_ and 5_3_, dorsal. Scale bars in micrometers.

Antennules distinctly 5-segmented, generally directed straight forward (Fig. 3A, B). Geniculation placed between fourth and fifth segment. Antennule total length 0.36 mm, representing 28.6% of total body length and 82.8% of cephalothorax length. Relative length of five antennular segments (as % of total antennule length) as 15.2: 20.2: 19.3: 21.5: 23.7. First segment armed with spine 1 on inner distal part, arising slightly dorsally. Second segment armed with five spines (2v_1–3_, 2d_1, 2_) and strap-like, bipinnate seta IId: ventral spines (2v-series) subequal in length, 2d_1_ shorter than ventral counterparts; long spine 2d_2_ reaching midway of fourth segment, setiform and bipinnate with fine setules. Third segment with three elements: spine 3 located distomedially, IIId, IIIv setae bipinnate, disposed medially on segment. Fourth segment with eight elements (4v_1–3_, 4d_1, 2_, 4a, IVv, 4aes): spines 4v_1, 2_ robust, pinnate; spine 4v_3_ naked, slightly longer than former ones; naked spines 4d_1_ and 4d_2_ slightly thinner and shorter than those of 4v-series; spine 4a relatively well developed, subequal in length to members of 4d-series; seta IVv and ventral aesthetasc 4 (4aes) arising on proximal half of segment. Distomedial margin of segment fringed with hyaline frill. Fifth segment armed with 12 elements: short apical aesthetasc (5aes) arising near tip; three spines (5_1–3_) placed on distal part of segment (spine 5_1_ located distally, naked; 5_2_, 5_3_ located dorsally and slightly longer than 5_1_, bipinnate with fine setules (Fig. 3C)); distolateral margin of segment without branched setae, which are replaced with four well-developed, simple setae A–D, and with two short, thin simple setae a and b; ventral seta (Vv) arising midway of segment; minute spine 5a located proximally on inner margin; distomedial margin of segment with five transverse serrate ridges, proximal part of medial margin with hyaline frill.

**Figure 4.**
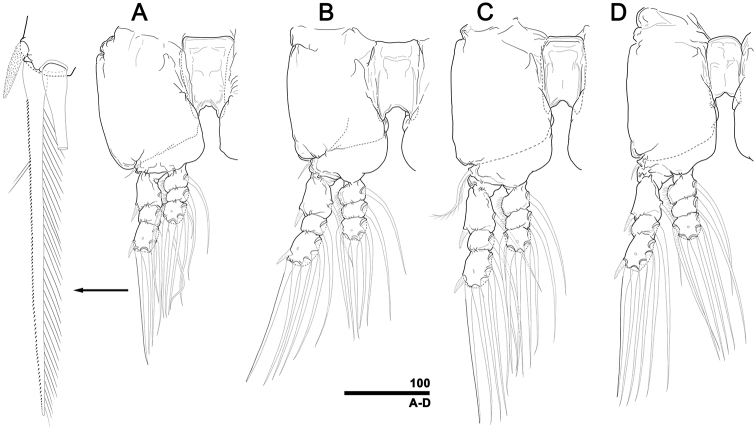
*Caromiobenellaohtsukai* sp. n., male holotype (MABIK CR00244260), swimming legs with intercoxal sclerites **A** Right leg 1 with inset of outermost seta of third exopodal segment (arrow), anterior **B** Right leg 2, anterior **C** Right leg 3, anterior **D** Right leg 4, anterior. Scale bar in micrometers.

First pedigerous somite (incorporated to cephalothorax) and three succeeding free pedigers each with pair of well-developed legs (Fig. 4A–D). Each protopod consisting of large, square coxa and relatively small basis. Border between coxa and basis on anterior face incompletely defined, but posterior diagonal articulation clearly expressed. Coxae of each leg pair joined by long, rectangular intercoxal sclerite, its length in legs 1–4 respectively 1.4, 1.5, 1.9, and 1.7 times longer than corresponding proximal width (mean = 1.6). Distal margin of intercoxal sclerites excavated (Fig. 4A–D). Basis of legs 1, 2 and 4 with short, simple seta proximally on outer margin, barely reaching to proximal part of first exopodal segment; this seta on leg 3 coarsely plumose, much longer, reaching to end of first exopodal segment. Both exopod and endopod 3-segmented; endopod always located anteriorly to exopod. Endopod of all legs shorter than exopod, reaching distal margin of second exopodal segment. First and third exopodal segments twice longer than corresponding second segment. All endopodal segments subequal in length, outer margin of first two segments fringed with fine hairs. Setal armament pattern of legs 1–4 as follows (Roman numerals indicate number of spines, Arabic numerals indicate number of setae):

**Table:** 

	Coxa	Basis	Exopod	Endopod
Leg 1	0-0	1-0	I-1; 0-1; I, 2, 2	0-1; 0-1; 1, 2, 2
Legs 2–4	0-0	1-0	I-1; 0-1; I, 2, 3	0-1; 0-1; 1, 2, 2

Spines on first and third exopodal segment pinnate, outermost seta on third exopodal segment serrate along outer margin, while pinnate along inner margin (Fig. 4A). Other setae subequally long except for relatively short inner setae on first exopodal segments: latter reaching end of exopods. Last segment of each ramus with pore on anterior face. Fifth legs knob-like, devoid of setae, reduced, implanted posteroventrally on first urosomal somite (Fig. 2C, D).

Genital somite with well-developed genital field on ventral side, composed of robust genital shaft plus two short, subtriangular lappets (Fig. 2C, D). Shaft protruding at midpoint along anterior face (Fig. 2D), then tapering distally. Hand-like opercular flap located on distal part at each side of genital shaft. Posterodistal part of genital shaft with prominent, smooth medial protrusion.

Caudal rami diverging from posterior margin of anal somite, each ramus 0.07 mm long, 0.04 mm wide, armed with six setae distributed as follows (Fig. 2C): two on outer lateral side (II, III), one terminally (IV), two on inner terminal corner (V, VI), and one on posterodorsal surface (VII). Setae II–VI subequal in length. Dorsal seta VII noticeably shorter than rest. All caudal setae bipinnate. Pore present on posterior ventral surface of each ramus.

######## Etymology.

The species name is dedicated to Prof. Susumu Ohtsuka (Hiroshima University, Japan) for his remarkable contributions to copepod taxonomy and ecology.

######## Remarks.

The present male specimens are assignable to the genus *Caromiobenella* based on the display of the generic features of males proposed by Jeon et al. (2018a): a relatively short cephalothorax not exceeding half of total body length, antennules with a modified fifth segment with the inner distal part bearing five serrate transverse rows of spinules, an inconspicuous oral papilla, a genital apparatus consisting of a robust shaft and two short, subtriangular lappets, and specific ornamentations such as two pairs of prominent crater-like depressions anteriorly and two longitudinal rows of four pores each posteriorly on the dorsum of cephalothorax. Besides such consistent features through the male congeners, some features are presented in two different ways, i.e. two types of male genitalia (McAlice 1985; Suárez-Morales 2000; Jeon et al. 2018a) and having five or six caudal setae (Jeon et al. 2018a). The combination of the latter two variations eventually leads the present species to be unique and differentiated from any other known male congener.

Two types of male genitalia have been reported to occur in this genus (McAlice 1985; Suárez-Morales 2000; Jeon et al. 2018a): one with the genital shaft displaying a deep triangular notch on the posterodistal margin (type I hereinafter), and a second one with a smooth medial protrusion instead of a notch in homologous position (type II hereinafter). This differential criterion divides the *Caromiobenella* species into two subgroups, with those sharing type I genitalia represented by *C.castorea* including *C.helgolandica*, *C.pygmaea* and *C.patagonica*, whereas type II genitalia is displayed by *C.polluxea* including *C.serricornis*. Only two species remain to be accommodated in this framework: *C.hamatapex*, whose type of male genitalia has not been reported thus far, and *C.arctica*, whose male genitalia were not described with enough detail. The present species displays type II genitalia and thus could be more closely related to the *C.polluxea* species-group.

Furthermore, members of *Caromiobenella* can also be divided into another two subgroups based on the display of five or six caudal setae (Jeon et al. 2018a). Thus, the *C.castorea* species-group display six caudal setae and includes *C.helgolandica* sensu McAlice, 1985, *C.arctica*, *C.hamatapex* and *C.patagonica*. The *C.polluxea* species-group displays only five and includes *C.helgolandica* sensu Huys & Boxshall, 1991, *C.serricornis* and *C.pygmaea*. With respect to the number of caudal setae, the present new species can falls in the *C.castorea* subgroup.

The combination of type II genitalia and six caudal setae (presented in form of “II-6” hereinafter) makes the new species described herein unique, as the rest of congeners known hitherto present a different combination of these two features: I-6 for *C.castorea*, II-5 for *C.polluxea*, I-5 or I-6 for *C.helgolandica*, II-5 for *C.serricornis*, I-5 for *C.pygmaea*, and I-6 for *C.patagonica* (Claus 1863; Sars 1921; McAlice 1985; Huys and Boxshall 1991; Suárez-Morales 2000; Suárez-Morales et al. 2008; Jeon et al. 2018a).

The monospecificity of *Caromiobenellahelgolandica* has been frequently questioned (Grygier and Ohtsuka 1995, Suárez-Morales 2010, 2011). McAlice (1985) considered *Monstrillacanadensis* as the male of *M.helgolandica* (= *C.helgolandica*; Jeon et al., 2018a). This author provided three illustrations of male caudal rami (McAlice 1985: fig. 1, 1–3c) where five caudal setae are clearly shown, but remarked in the main text that one more additional short dorsal seta occurred as well, although it was difficult to observe. In addition, the author also mentioned that the general setation pattern of the male caudal rami was the same as in the female, which is depicted with six caudal setae (McAlice 1985: fig. 2f). In referring to these two descriptions, we understand McAlice’s *C.helgolandica* specimens have six caudal setae. On the contrary, Huys and Boxshall (1991: fig. 2.5.8a, b) depicted the same appendage as bearing only five caudal setae without any accompanying descriptive text. Despite such morphological discrepancy by different authors and the uncertainty on the actual identities of those taxa, the present new species is clearly distinguished from the previously known *C.helgolandica* (or *C.helgolandica* species-complex) by type of the genitalia. The illustrations of male genitalia by McAlice (1985: fig. 1, 1–3d) showed a specific deep notch, and those by Huys and Boxshall (1991: fig. 2.5.8b) were depicted without a medial protrusion as prominent as in *C.polluxea*, *C.serricornis*, or in the present new species. Another presumed congener *C.arctica*, originally reported from the Arctic region (Resolute Bay, Cornwallis Island, Canada), was originally described without including detailed information on its male genital apparatus. This species, however, can be differentiated from the present new species by the display of unusual features such as an anterior rostral projection on the cephalothorax and the presence of three dichotomously branched setae on the last antennular segment (Davis and Green 1974).

*Caromiobenellahamatapex* is known only from the female; thus, direct comparison with the present male specimens is risky due to the occurrence of strong sexual dimorphism in the order Monstrilloida (Suárez-Morales 2007; Suárez-Morales et al. 2008; Jeon et al. 2018b).

One of the most important morphological key features for the recognition of the new species is the presence of a fifth leg reduced to a small, knob-like, rudimentary protuberance. The absence of the fifth legs in males is one of the diagnostic characteristics for this genus (Sars 1921; Davis and Green 1974; McAlice 1985; Huys and Boxshall 1991; Suárez-Morales 2000; Suárez-Morales et al. 2008; Jeon et al. 2018a) although some specimens of *C.polluxea* have been reported to display a unilateral small nipple-like structure on the ventral side of the first urosomal somite (see Jeon et al. 2018a). Unlike in *C.polluxea*, a fifth pair of legs was present in all the type specimens examined; thus, it can be regarded as one of the genuine features for this species, and this feature separates it from the rest of representatives of the genus.

### Molecular analysis

Portions of the mtCOI and 28S rRNA genes were sequenced for five male *Caromiobenellaohtsukai* sp. n. individuals. Excluding the primer binding sites, 520 and 782 base pairs (bp) were sequenced for mtCOI and 28S rRNA, respectively. The average GC content was 30.4% for mtCOI and 51.4% for 28S rRNA. Sequences from the five *C.ohtsukai* sp. n. specimens were aligned with six additional GenBank sequences from *Caromiobenella* (three sequences), *Monstrilla* (two sequences), and *Lepeophtheirussalmonis* (Copepoda, Siphonostomatoida) as an outgroup (Table 1).

**Table 1.** List of specimens used for molecular analysis, specimen voucher information, and GenBank accession numbers for mtCOI and 28S rRNA sequences.

Species	Specimen voucher	Accession number	
		mtCOI	28S rRNA
*Caromiobenellaohtsukai* sp. n.	HYU-Mon0048	**MH638357**	**MH647065**
*Caromiobenellaohtsukai* sp. n.	HYU-Mon0049	**MH638358**	**MH647066**
*Caromiobenellaohtsukai* sp. n.	HYU-Mon0050	**MH638359**	**MH647067**
*Caromiobenellaohtsukai* sp. n.	HYU-Mon0051	**MH638360**	**MH647068**
*Caromiobenellaohtsukai* sp. n.	HYU-Mon0052	**MH638361**	**MH647069**
* Caromiobenella castorea *	HYU-Mon0001	KY553209	KY563281
* Caromiobenella polluxea *	HYU-Mon0006	KY553211	KY563286
* Caromiobenella hamatapex *	LEGO-MON005	KR048994	KR048920
* Monstrilla ilhoii *	HYU-Mon0009	KY553214	KY563289
*Monstrilla* sp.01	HYU-Mon0024	KY553220	KY563303
* Lepeophtheirus salmonis *	LEGO-SIP012	KR049052	KR048867

Accession numbers for newly obtained sequences in bold.

Mitochondrial COI sequences used in the multi-species alignment were 480 bp in length containing 267 (55.6%) polymorphic sites and 165 (34.4%) parsimony-informative sites. The genetic mtCOI mean divergence within the individuals of the new species was 0.76% (0.21–1.26%), and the mean between-species divergence within *Caromiobenella* was 21.47% (20.16–23.12%). The mean divergence between *Caromiobenella* and *Monstrilla* species was 36.57% (34.11–40.48%). All 28S rRNA sequences were aligned in the same manner. The alignment included 757 bp for all sequences. Of these 757 sites, 323 (42.7%) were variable and 188 (24.8%) were parsimony-informative. There was no genetic difference (0.00%) between the five sequenced specimens of the new species. The mean divergence between species of *Caromiobenella* was 12.66% (7.81–14.61%), and the mean divergence between *Caromiobenella* and *Monstrilla* species was 27.32% (26.23–28.81%). These results confirm that previously described *Caromiobenella* species are genetically distinct from each other, and that between-genus genetic differentiation is greater than between-species differentiation within *Caromiobenella*. Substitution saturation tests indicated little saturation in the present sequence datasets.

## Discussion

**Figure 5.**
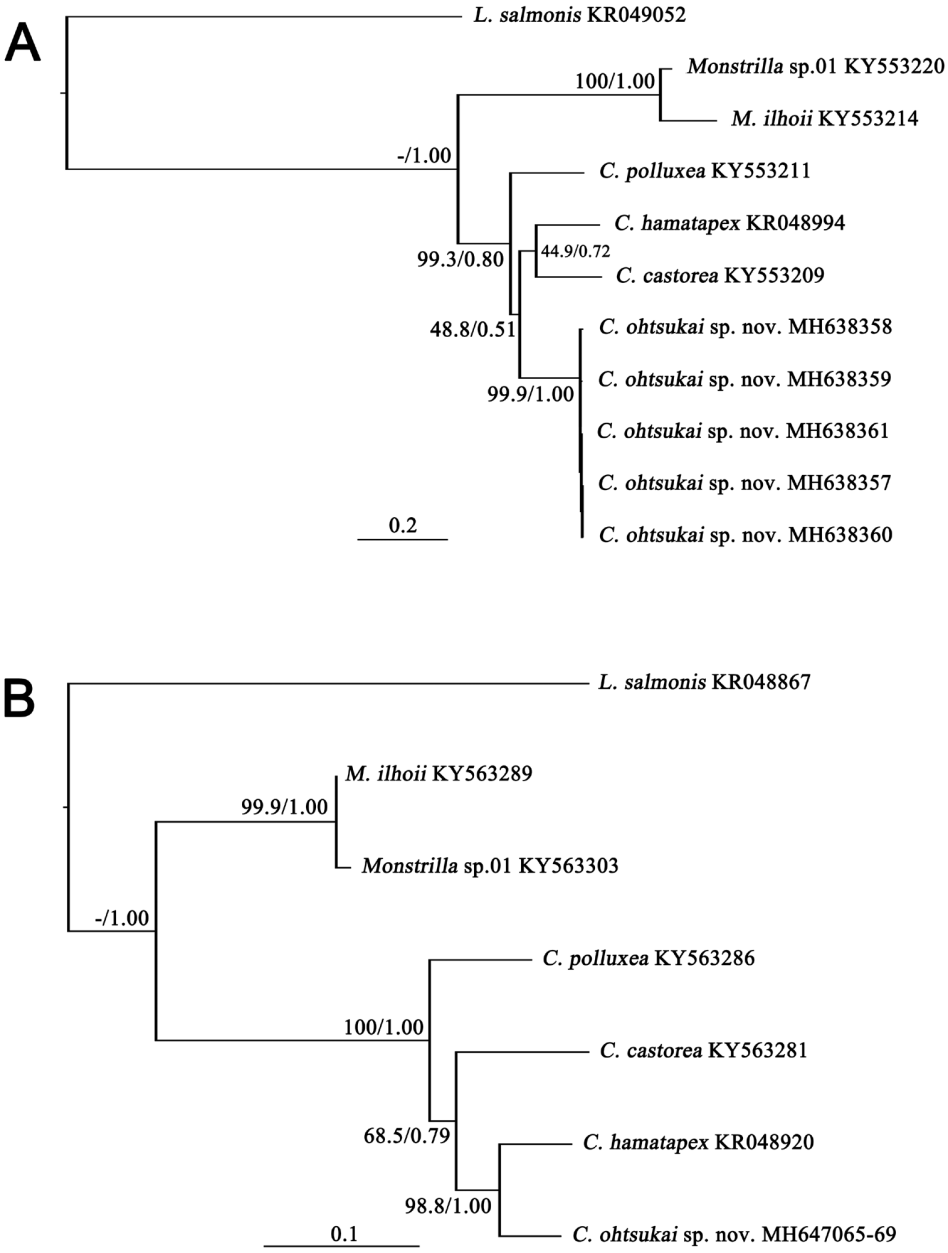
Phylogenetic trees reconstructed based on the sequences derived from two genera and six species of monstrilloids including an outgroup taxon, *Lepeophtheirussalmonis* (Siphonostomatoida). The numbers above or below branches indicate both bootstrapping values (BP, in percentage) and Bayesian Posterior Probabilities (BPP, in probability) and are presented in order of BP/BPP **A** Tree based on the sequences of mtCOI **B** Tree based on the sequences of 28S rRNA. Each species name followed by the GenBank accession number(s).

**Figure 6.**
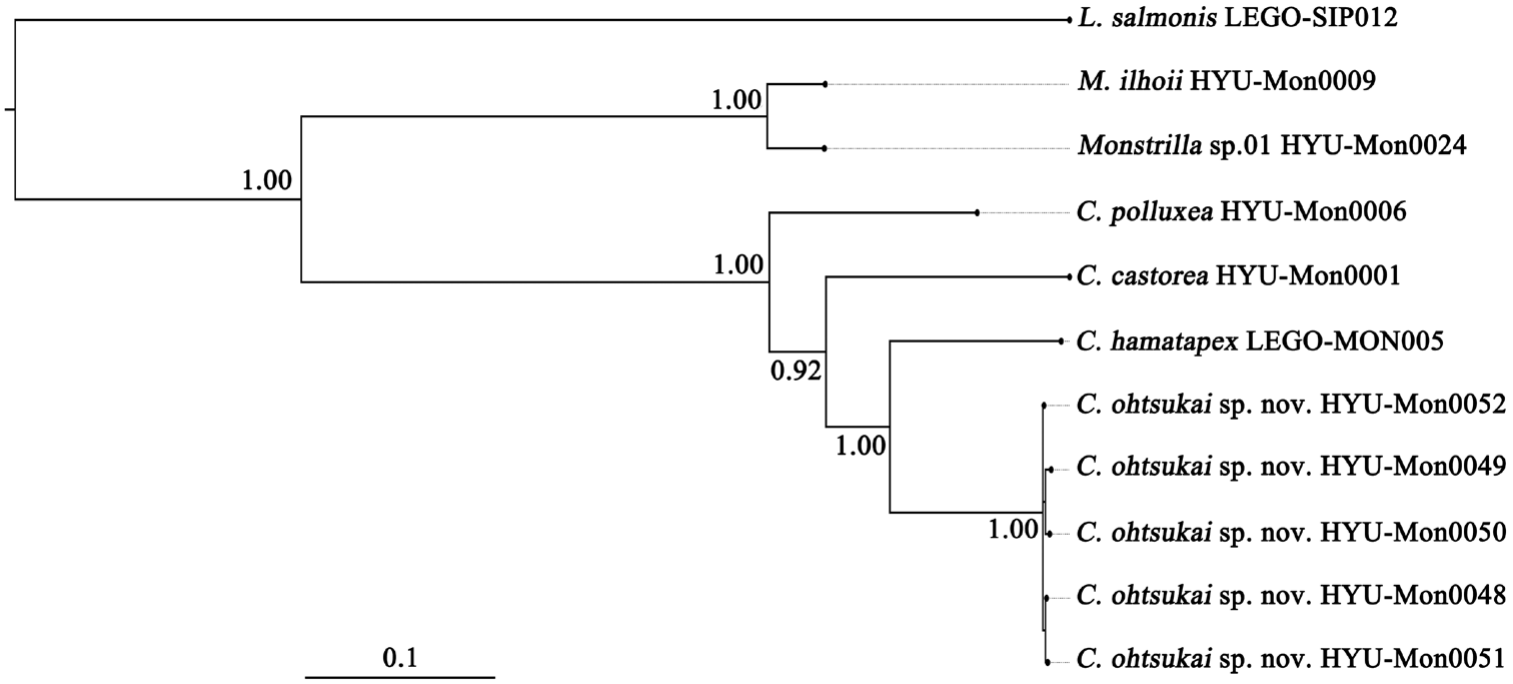
Phylogenetic tree reconstructed based on the concatenated sequences consisting of mtCOI and 28S rRNA. The numbers above or below branches indicate Bayesian Posterior Probabilities. Each species name followed by the specimen voucher registered in the GenBank.

The molecular analysis presented herein supports the conclusion that the new species is distinct from other congeners including *Caromiobenellahamatapex*. Molecular data compensate for the lack of morphological information. Genetic divergence between the new species and *C.hamatapex* based on mtCOI sequences was 20.8%, similar to the mean between-species divergence (21.5%) found in *Caromiobenella* species. These values exceed the known species delimitation threshold of 10–15% divergence. Hebert et al. (2003) estimated that a 10% genetic divergence threshold is required for mtCOI sequences to indicate species differentiation in congeners. In that study, however, the majority of crustaceans showed even higher divergence levels (16–32%; mean = 15.4%, *N* = 1,781). Lefébure et al. (2006) proposed a similar molecular threshold for species delimitation (0.16 nucleotide substitution rate per site). Using these previously determined thresholds for divergence and considering similar values for other Copepoda groups (Blanco-Bercial et al. 2014; Baek et al. 2016; Krajíček et al. 2016; Bradford-Grieve et al. 2017), the results presented here indicate that the taxon we describe differs from *C.hamatapex* at the species level. Jeon et al. (2018a) calculated about 10% genetic divergence based on 28S rRNA among congeneric species of monstrilloids and a twofold higher value for between-genus divergence. In that study, the two genera *Caromiobenella* and *Monstrilla* had a 26.67% genetic divergence for 28S rRNA. Analyses presented here resulted in a similar degree of genetic divergence (27.23%), supporting the conclusion that the two genera are distinct from each other. Clear separation of the two genera was confirmed in both ML and BI trees based on mtCOI and 28S rRNA sequences with high branch supporting values (Fig. 5). Additional BI analysis based on the concatenated dataset clarified taxonomic separations at both species and genus levels with robust branch supporting values (Fig. 6).

## Supplementary Material

XML Treatment for Caromiobenella ohtsukai
